# A developing country response to Lavery *et al*. "In global health research, is it legitimate to stop clinical trials early on account of their opportunity costs?"

**DOI:** 10.1186/1472-6939-10-16

**Published:** 2009-09-30

**Authors:** Douglas R Wassenaar, Gita Ramjee

**Affiliations:** 1 Biomedical Research Ethics Committee, University of KwaZulu-Natal, Durban, South Africa; 2 South African Research Ethics Training Initiative (SARETI), University of KwaZulu-Natal, Pietermaritzburg, South Africa; 3 HIV Prevention Research Unit, Medical Research Council, Durban, South Africa

## Abstract

**Background:**

A recent paper presents an argument and mechanism for the possible stopping of clinical trials early based on opportunity costs.

**Discussion:**

Although we agree that the costs and opportunity costs of clinical trials need to be reduced wherever possible, we raise concerns about the motivation and mechanism for stopping clinical trials early raised by Lavery *et al*.

**Summary:**

We argue that there are already enough acceptable criteria and actors in the clinical trials arena to justify early stoppage of clinical trials, and argue that factors other than efficacy need to be carefully considered, especially in developing country contexts.

## Background

In response to recent arguments for a mechanism to stop certain clinical trials early based on 'opportunity costs', Lavery *et al*. [1] develop some arguments and a mechanism for doing so, so that the resources saved can be redirected to more promising products. In the same paper, Buchanan raises some critical concerns about these proposals.

## Discussion

Although we agree with many of the points, particularly the reduction of opportunity costs in clinical trials, raised by Lavery *et al*. [1], we are largely more persuaded by Buchanan's views, to which we wish to add two more discussion points.

Firstly, as an IRB chair and microbicide trials PI respectively from a developing country setting, we have concerns about the introduction of yet another player, the proposed Scientific Oversight Committee (SOC) into the already complex clinical trials oversight arena. At present clinical trials are subject to oversight and amendment by sponsors, IRBs, regulatory authorities, host institutions, government health departments, DSMBs and various layers of community input and participation. While essential to the ethical and scientific conduct of a clinical trial, engagement and compliance with these parties has various financial, personnel and efficiency costs. Introduction of yet another player will only further increase this 'administrative drag'. The costs of this additional layer need to be factored against any proposed advantages.

A completed Phase III randomised clinical trial can yield unequivocal efficacy data. Clinical trials with negative outcomes yield important scientific information. The Col 1492 study data on a failed microbicide [2,3] has yielded unequivocal data, making it clear that that particular product line is not worth further investment and risk. However, the scale of the HIV pandemic in Africa warrants the development of products that might have partial efficacy that will nevertheless have significant public health impact. For example, condoms are known to be highly efficacious in preventing HIV transmission, but for social and cultural reasons their uptake in the real world remains low [4,5], having only a minor impact on reducing transmission of HIV. In contrast, a potential product with lower efficacy but higher community uptake and acceptability could have a larger public health impact on reducing HIV transmission. Our point is that the HIV prevention field at present would benefit from a spectrum of interventions, all of which require completed Phase III data to support their use, even if efficacy is lower than that expected of newer products in the design pipeline. Product effectiveness is not narrowly tied to efficacy, as is well known. A less efficacious product may have the greatest *public health benefits*, as the relative failure of condoms and VCT have shown [4,6,7]. The prevention field needs multiple interventions. These will vary in efficacy but maximise effectiveness.

Our second point is that clinical trials conducted in developing country settings involve significant investments in scientific and community capacity building. A narrow focus on the product itself, or its opportunity costs, overlooks the impact of stopping a clinical trial early (for other than efficacy, safety or futility reasons) on the morale and development of the participating scientists and related personnel, and on the host community. Furthermore, we cannot switch products without scientific data. Prior to commencement of the trial, participants and participating communities are informed of preclinical evidence of a particular product and the reasons why the product should undergo large scale human testing. If the product is then deemed less promising based on a newer product, the community will be justified in asking why the research was commenced in the first place if the scientist did not feel confident about its potential efficacy. In addition, what scientific justification will be used to argue that the newer product is going to be more efficacious? Pre-clinical data or early safety testing? Even if the community is forewarned on the possible switch, it does not negate enormous concern about the likely distrust the process may create. There is likely to be a temporal gap in switching products as it will involve new protocol, new approvals, training of staff (if we are able to retain the team) and re-educating the community.

Clinical trials are rightly increasingly expected by developing country IRBs and international ethics guidance to have a community engagement [8] and capacity building agenda [9,10]. The abrupt stoppage of a trial by a SOC because a more promising product is on the horizon does not take into account the emotional and moral commitment of communities to a particular study process. It is a difficult enough process to engage with participating communities when studies are stopped for safety or futility reasons, as the recent HIV vaccine STEP and Phambili study stoppages have shown [11,12]. The first author, as an IRB chair, has already had to intervene on behalf of an investigator whose sponsors were considering discontinuing his study, in its existing design, based on data from other studies which had shown some efficacy. These reference studies, however, were related but not identical with regard to the intervention itself, the controls, or, importantly, socio-cultural context. In this case not even DSMB analyses were referenced. These grounds were disputed by the trial team. It seemed clear that the sponsor wanted to divert funds to an apparently more promising new product - i.e., because of perceived opportunity costs. No consideration was apparently given to the developmental investment by the host institution, the scientific and technical staff, or the participating community. The study was eventually allowed to continue.

We are concerned that the abrupt withdrawal of studies by a proposed SOC will erode community engagement in clinical trials in general, even if the role and potential impact of SOC decisions are added to the (already complex) enrolment and consent process. The loss of community support and confidence in clinical trials can constitute an opportunity cost not considered by Lavery *et al*. or Buchanan. It is interesting to note that the decision tree proposed by Lavery *et al*. bypasses IRBs, whose primary purpose it is to protect the welfare and dignity of participating individuals and communities. IRBs are merely to be 'advised' of study terminations by the SOC.

While the HIV prevention field needs innovation, and effective new products are sorely needed, we argue that current mechanisms are the only ones that should be used to determine whether a clinical trial should be stopped early. Due consideration should also be given to development agendas and the obligations of the study site to the participating communities whose engagement and investment reflect more than efficacy concerns. The costs of a cold-blooded SOC decision to stop a trial will indeed be "measured in the lives of the poor" [1].

We do, however, agree that investment in clinical trial site development needs to be sensitive to opportunity and other costs, and that trial sites should consider resource sharing to accommodate initiatives and activities that can make more efficient use of scarce resources, especially in developing country settings. This can and should be done without the oversight of an SOC. For example, many initiatives and centres in Africa are disease-specific, and increased consideration should be given to breaking these expensive and confining 'silos' and expanding the spectrum of diseases targeted by such resource-intensive research centres [13].

Premature stoppage of a trial for other than efficacy, safety or futility reasons seems to us not to be a viable option. The introduction of a SOC and implementation of its decisions may have major opportunity costs of its own. Furthermore, the role of a SOC would be more suited at the product selection process and not after the trial has been implemented. Rather, sites with clinical trial capacity should endeavour to share resources (skills, laboratory capacity, community engagement efforts) to reduce the opportunity costs of a single trial with a long-term developmental agenda in mind. We argue that this can and should be done without the intrusion of an SOC or expanded DSMB.

## Summary

We argue that clinical trials, especially in the HIV prevention field, should only be stopped early on safety or futility grounds, and that developing country trial site capacity and community trust in trials could be eroded by stoppage on other grounds and mechanisms. We further argue that the HIV prevention field will probably require many products of varying efficacy until such time as an effective preventive technology is in place.

## Abbreviations

IRB: Institutional Review Board; DSMB: Data Safety Monitoring Board; VCT: Voluntary Counselling and Testing.

## Competing interests

The authors declare that they have no competing interests.

## Authors' contributions

DW initiated and drafted the article and GR added arguments, comments and examples and assisted the first author with the final review of the paper. Both authors read and approved the final manuscript.

## Authors' information

DW is Principal Investigator of the South African Research Ethics Training Initiative (SARETI) funded by NIH/Fogarty International Center grant no. 5 R25 TW001599-10 which partially funded the work on this paper, and is Chair of the University of KwaZulu-Natal Biomedical Research Ethics Committee.

GR is the director of the HIV Prevention Research Unit of the South African Medical Research Council in Durban. She was the principal investigator of most of the Microbicide trials conducted to date.

## Pre-publication history

The pre-publication history for this paper can be accessed here:


